# Rwanda’s evolving community health worker system: a qualitative assessment of client and provider perspectives

**DOI:** 10.1186/1478-4491-12-71

**Published:** 2014-12-13

**Authors:** Jeanine Condo, Catherine Mugeni, Brienna Naughton, Kathleen Hall, Maria Antonia Tuazon, Abiud Omwega, Friday Nwaigwe, Peter Drobac, Ziauddin Hyder, Fidele Ngabo, Agnes Binagwaho

**Affiliations:** School of Public Health, University of Rwanda College of Medicine & Health Sciences, Kigali, Rwanda; Ministry of Health, Kigali, Rwanda; Partners In Health/Inshuti Mu Buzima, Rwinkwavu, Rwanda; World Bank, Kigali, Rwanda; UNICEF, Kigali, Rwanda; Division of Global Health Equity, Brigham and Women’s Hospital, Boston, 02115 MA, USA; Department of Global Health and Social Medicine, Harvard Medical School, Boston, 02115 MA USA; Division of Global Health and Social Medicine, Ministry of Health, Kigali, Rwanda; Harvard Medical School, Boston, 02115 MA USA; Geisel School of Medicine, Dartmouth College, Hanover, 03755 NH USA

**Keywords:** Rwanda, CHW, Health system, Provider and Clients’ perspectives, Qualitative assessment

## Abstract

**Background:**

Community health workers (CHWs) can play important roles in primary health care delivery, particularly in settings of health workforce shortages. However, little is known about CHWs’ perceptions of barriers and motivations, as well as those of the beneficiaries of CHWs. In Rwanda, which faces a significant gap in human resources for health, the Ministry of Health expanded its community health programme beginning in 2007, eventually placing 4 trained CHWs in every village in the country by 2009. The aim of this study was to assess the capacity of CHWs and the factors affecting the efficiency and effectiveness of the CHW programme, as perceived by the CHWs and their beneficiaries.

**Methods:**

As part of a larger report assessing CHWs in Rwanda, a cross-sectional descriptive study was conducted using focus group discussions (FGDs) to collect qualitative information regarding educational background, knowledge and practices of CHWs, and the benefits of community-based care as perceived by CHWs and household beneficiaries. A random sample of 108 CHWs and 36 beneficiaries was selected in 3 districts according to their food security level (low, middle and high). Qualitative and demographic data were analyzed.

**Results:**

CHWs were found to be closely involved in the community, and widely respected by the beneficiaries. Rwanda’s community performance-based financing (cPBF) was an important incentive, but CHWs were also strongly motivated by community respect. The key challenges identified were an overwhelming workload, irregular trainings, and lack of sufficient supervision.

**Conclusions:**

This study highlights the challenges and areas in need of improvement as perceived by CHWs and beneficiaries, in regards to a nationwide scale-up of CHW interventions in a resource-challenged country. Identifying and understanding these barriers, and addressing them accordingly, particularly within the context of performance-based financing, will serve to strengthen the current CHW system and provide key guidance for the continuing evolution of the CHW system in Rwanda.

## Background

Shortages of qualified human resources for health are particularly acute in low-income countries, which face significant burdens of disease and mortality. In 2010, 7.6 million children died before reaching their fifth birthday worldwide, and in Africa, almost three fourths (73.2%) of the under-5 mortalities were due to preventable infectious causes, including pneumonia, diarrhoea, and malaria [1]. Skilled health worker shortages are among the reasons that impede access to treatment, but it has been found that effective and inexpensive interventions for these diseases can be delivered by community health workers (CHWs) [2].

CHWs are increasingly being utilized to alleviate the shortage of human resources for health, particularly in the delivery of interventions for maternal and child health [3]. By serving as intermediaries between the community and the formal health system, CHWs are uniquely placed to serve resource-poor settings. CHW systems have been found to be effective in implementing interventions to prevent under-five deaths, including malaria prevention, health education, breastfeeding promotion, essential newborn care and psychosocial support [4]. When health centres offer the minimum package of services, CHWs' services can support the continuum of care and provide valuable referral health services [5, 6].

Effective CHW case management has been linked to the level of training, transport means, adequate supervision, access to medicines and educational tools, and sufficient incentives [7–11]. Barriers, however, can reduce effectiveness of CHW service delivery, including inadequate training, supervision, and remuneration, as well as insufficient assimilation of CHWs into the health system [12, 13].

Rwanda is one of the only countries in the sub-Saharan region on track to achieve the health-related millennium development goals by 2015 [14]. Between 2000 and 2011, under-5 mortality rates decreased by 70.4%, and Rwanda experienced an average annual rate of reduction (AARR) of 5.1% in the under-5 child mortality rate between 1990 and 2011 [15]. The AARR between 2000 and 2011 was 11.1%, which was the world’s highest decrease during this time frame [14]. The maternal mortality ratio in Rwanda has been estimated at 340 deaths per 100,000 live births in 2010 and a decrease of 59.5% was observed from 2000 to 2010 [15].

Despite these impressive health gains, the country still faces significant challenges, including one of the most severe shortages of human resources for health on the continent [16]. Even with the remarkable advances in premature mortality in the recent decade, the under-5 mortality rate is 76 per 1,000 live births, with 1 in 13 children born in Rwanda still dying before reaching their fifth birthday. The main causes of death include diarrhoea, pneumonia, and HIV/AIDS, among others. Populations in remote areas, or those particularly affected by financial insufficiency are most at risk [17].

Aiming to overcome these health burdens, in 2007 Rwanda initiated a reform of the national community health system, which was initially implemented in 1995. In this system, CHWs are required to have a minimum of 6 years of education, and are elected by their communities. At the time of this study, there were approximately 60,000 CHWs in Rwanda, comprising 3 CHWs per village. Each village had a pair of general CHWs (called a *binome*) who were responsible for community health, nutrition, and HIV/AIDS prevention, and a maternal health worker (referred to as an *Animatrice de Santé Maternelle* (ASM)), who managed infant, and pre and postnatal maternity care. In addition, each village had a CHW in charge of social affairs (CHSA) dedicated to addressing the well-being of individuals and the community [18].

This study aims to qualitatively evaluate the CHW system in Rwanda from the perspective of CHWs themselves, as well as their beneficiaries. Moreover, the study seeks to identify factors and challenges that impact the CHW system and can inform system improvements.

## Methods

### Study design and population

A cross-sectional descriptive study was conducted to collect qualitative information regarding educational background, knowledge, and practices of CHWs, as well as the perception of CHWs from the beneficiaries, including women of reproductive age. Focus group discussions (FGDs) for both CHWs and their beneficiaries were conducted, which included the collection of demographic data. These data were collected as part of a larger assessment of CHW capacity relating to community-based nutrition (CBN) programmes, as commissioned by the Rwanda Ministry of Health (MoH) with the support from World Bank and UNICEF Rwanda.

The study was held from 10 to 20 May 2011, in 3 districts with differing levels of food security, which was selected in accordance with the overall goal of the CHW CBN capacity study. District selection was based on assessment of food consumption levels as reported in the 2009 Rwandan Comprehensive Food Security and Vulnerability Analysis and Nutrition Survey (CFSVA). The CFSVA report divided all districts into three categories of food consumption (high, moderate, and low). One district from each of these categories was purposively selected: Gicumbi with a food consumption score above the national average; Kamonyi with a moderate food consumption score; and Ngororero with a poor food consumption score.

In each of the districts, two administrative sectors were selected via simple random sampling (using the EPIINFO randomization programme). In one sector per district, one focus group composed of twelve *binomials*, and one focus group of woman beneficiaries of CBN services (twelve women of reproductive age, including at least four pregnant women and four lactating women) was organized. Women are the primary beneficiaries of the CBN services offered by the CHWs, and because vaccination uptake is 98% and antenatal care is sought by over 90% of women [17], this approach minimized potential selection bias.

In the other sector selected in the district, a focus group was organized for 12 ASM, and 12 CHSA CHWs. The individual CHWs and beneficiaries were selected randomly, again with simple random sampling, from lists furnished by the local health centres. Thus, in each of the 3 districts, 108 CHWs (12 CHWs per category - ASM, *binomes*, and CHSA) and 36 beneficiaries (12 per district) were included in FGDs. No CHWs or beneficiaries declined to participate in the FGDs.

### Data collection

Focus group guides for beneficiaries and CHWs were developed following meetings with Government of Rwanda health officials, pretested and translated into the local language of Kinyarwanda. The FGD guides included demographic data. Data were collected over a 10-day period in May 2011. The FGDs were conducted by experienced and trained enumerators, and were audio-recorded, transcribed in the local language of Kinyarwanda, and then translated into French by a professional translator.

### Data processing and analysis

The translated transcripts collected from the field were assessed by two coder clerks who identified emergent codes from the FGDs. Once the final set of codes was agreed upon, they were analyzed using qualitative analysis software, Atlas.ti. Beneficiaries’ demographic characteristics, CHWs’ demographic characteristics and educational background were also recorded.

### Ethical considerations

Authorization from the MoH was secured before data collection. A written request from the Maternal and Child Health Department under the MoH was submitted to district hospitals and administrative managers at the district level. Informed consent forms were read aloud to the group members of the FGD and were obtained before starting the interview. Participants were assured that all responses were anonymous, and not identifiable to any individual participant.

## Results

### Participants’ profile

A total of 108 CHWs (CHSA, ASM and *binome*), and 36 beneficiaries (lactating women, pregnant women and women of reproductive age) were interviewed for this study.

The median age (interquartile range (IQR) of CHWs was 38 (33 to 45) with 91% being married, 4% single and the remainder either widowed or divorced. Twenty-six percent of the CHWs were male. The median years of experience were 3, although this ranged from 1 to 16. The majority of the CHWs (76%) had only a primary level of education; the average number (±standard deviation (SD)) of children per CHW was 4 (±2.2) children.

Targeted beneficiaries of community services were generally younger than CHWs, with a median age of 24 (range, 19 to 45 years). Thirty-five (96%) of the beneficiaries were farmers, and 80% were married with an average (±SD) of 2 (±1.6) children per woman.

### CHW focus group discussions

CHWs identified five key areas that significantly influenced their effectiveness, including training, the role of community performance-based financing, supervision (cPBF), workload as perceived by CHWs, and motivations. In particular, findings relating to irregular trainings and confusion of the cPBF system were widespread among the FGDs, and constituted key findings of this study.

### Training

CHWs were generally selected by their communities based on criteria including literacy, past experience, and social acceptance. However, most of the CHWs did not have an education in health, and were not trained prior to beginning their roles as CHWs. Newly-recruited CHWs did not undergo an initial training programme, and only received on-the-job trainings. Although the importance of trainings was well known and widely recognized as key to improving knowledge, training sessions were found to be infrequent and not equitably delivered at the community level for all three types of CHWs.

For those who received trainings, the effectiveness of the programmes was highlighted, and CHWs claimed that the trainings helped to improve their knowledge and practices. Most CHWs appreciated the trainings, and felt that the community recognized and appreciated their advanced knowledge. However, trainings were described as inconsistent and insufficient, and some groups of CHWs did not attend any training programmes beyond the basic orientation programme.

The CHWs explained that *binomes*, as opposed to CHSAs and ASMs, received most of the trainings for nutrition, basic health, preventive, and curative services. One CHW said: 'We were not trained, but we can see what the *binomes* are doing in terms of benefits of training. We can see how they are taking care of malnourished children, but this does not have to do with our work'. Throughout the FDGs, CHSA and ASM CHWs highlighted this disparity in training, and noted their own lack of access to comprehensive training programmes. In regards to their role in promoting nutrition, many CHWs reported having had some training on nutrition, but the majority expressed the need to understand different components of a balanced diet.

While the national CHW policy does not intend for ASM CHWs to deliver infants, these CHWs said that they were often confronted with women delivering before arriving at the health centre. The lack of any training on delivery was considerably problematic and viewed as a gap in their knowledge ' … because we all know that the trainings will reinforce our capacity in reducing infant and maternal mortality', said one CHW.

The CHWs also explained that they lacked training on communication skills necessary to promote behaviour change in the community. The CHWs noted that their lack of confidence contributed to difficulties in convincing beneficiaries to adopt preferred behaviour such as preparing a balanced diet or practising basic hygiene. The lack of training in communication skills was seen as a complicating factor in facilitating promotion of behaviour change in the community.

### Performance-based financing

The Rwanda MoH uses community performance-based financing (cPBF), a remuneration mechanism based on outputs, to create incentives for CHWs. The CHWs are not provided with a monthly salary; however, CHWs are evaluated based on performance indicators and the resulting financial incentive is given to a cooperative of CHWs. Based on the FGD accounts, some CHWs did not clearly understand the concept of cooperatives or cPBF, and they had not received proper training on how to set up the financial mechanisms or manage the cooperatives.

According to the CHW respondents in this study, the attempts at including the individual CHWs in the management of cPBF have not been successful. Only one CHW spoke of the benefits of the CHW cooperative system, while some CHWs raised the point that transparency and accountability of cooperative managers contributed to hinder the financial success of their cooperatives. According to the respondents in the study, the workload required of CHWs was high, and for most CHWs, this engagement provided little or no financial gain. Some CHWs were unsure if the cPBF programmes were still ongoing, '… for the last 2 years, I do not know if the cPBF exists or if it was stopped', explained one CHW, and others misunderstood the system, assuming that every CHW would receive a dividend at the end of the year.

### Supervision systems

While national guidelines on CHW supervision existed at the time of this study, there were discrepancies in the frequency and content of supervision. Most CHWs met with their supervisors once a month at the health centre to deliver reports, although there were no standard procedures used for field supervision. Some supervisors accompanied CHWs on home visits occasionally, although this was not consistently a policy. The majority of CHWs recognized the importance of the supervision and noted this in the FGDs, explaining that, on occasion, they learned valuable information from supervision visits. However, CHWs often primarily associated supervision to the handover of the monthly report and not always to obtaining guidance and assistance.

### Workload

In terms of their workload, the CHWs considered their general range of duties to be overwhelming, and subsequently a barrier to the sustainability of the CHW system. The variable hours necessary for their work, and the unexpected crises that arose (such as epidemics), conflicted with CHW family life and their other jobs. The intensity of the work was explained as varied and unpredictable, and detracting from time necessary for families.

Many *binomes* felt that their range of work was too broad to allow a sufficient and quality provision of care. As a *binome* CHW explained, 'we have 255 households to follow up each month and each of us takes a quarter of the households to accomplish our work, to help each other'. It was found that as a result of the high workload, the three types of CHWs divided up the houses in the village, travelling only to the houses in their vicinity. This resulted in some households being visited by CHWs who did not have any training in specific areas, such as nutrition management or maternal health.

### Motivation

Despite an overwhelming workload, the CHWs experienced a sense of pride in their work. Many stated that they felt they were an important part of the whole health system improvement that aimed to reduce the burden of disease in the population. They recognized that under a decentralized system, their roles were increasingly becoming critical in reducing key health indicators, such as the infant and maternal mortality rates. By playing a direct role in improving indicators, CHWs felt valued and respected in their communities.

With regard to community recognition, almost all CHWs expressed their feelings about the improving health of the population: '… I feel happy when I see someone who was sick becoming healthy'. Relationships between themselves and their community fostered support and respect, as community members routinely approached the CHWs for advice. The ability to work for oneself, and manage one’s own hours, was also a clear incentive. Beneficiaries’ relatives also recognized the importance of CHWs in the community, as one CHW relayed what a man had said to her: 'without this programme, my wife would have died at home'.

A few CHWs also shared that the programme gave them a higher status despite their gender. Some claimed that having this position had given them more authority in the household and more autonomy over household purchases. Many of the ASMs who were trained on family planning had started using family planning methods themselves, and served as role models for the women in the community.

### Beneficiaries

For the women who participated in the community beneficiary FGDs, the majority made statements recognizing the benefits of the CHW system and a positive attitude towards the CHWs was generally noted. Multiple women expressed their appreciation for the CHW efforts to care for them and their families. One pregnant woman explained: 'I can see that all CHWs do the best they can … I can see that they are needed in our society', and another woman appreciated how 'they follow and support us on a daily basis'.

The beneficiaries described CHWs as their main source of educational messages about topics such as nutrition, malaria, kitchen gardens, family planning, and hygiene. Almost all beneficiaries in the focus groups seemed to know that when a child is malnourished, they should go to the CHW, and if the problem was serious, the CHW would accompany the child to the health centre and follow up on progress afterwards. They added that the CHWs explained the basics of nutrition to the families and made sure that the mothers understood. They further explained that they received frequent visits from CHWs at their houses (sometimes daily, weekly or twice a month) to check up on the health of the children and to teach them about healthy meal preparation. Most of them said that they would call the CHW or go to his/her house if they had a problem. Overall, the CHWs were perceived to be very influential in the lives of the women, with the beneficiaries only seeking out further care at the health centre for serious illnesses.

## Discussion

CHWs in Rwanda play key roles in linking the country’s primary health care system to the community, and mitigating the country’s health professional shortage by providing basic health care. While these intermediary positions are highly valued by the communities, CHWs experience a range of financial and capacity-building challenges that may limit their effectiveness and hinder integration with the formal health system. This study highlights the perceived benefits of the CHW system such as strong motivation and appreciation, as identified by CHWs themselves as well as their beneficiaries, in addition to the barriers that impede the effectiveness of the programme, including challenges of the cPBF system and irregular trainings.

CHWs interviewed in this study expressed a sincere desire to perform well, particularly because they felt valued by their colleagues and their communities, and perceived themselves as key assets to directly improving their communities’ health. While motivated primarily by community recognition and respect, opportunity costs impeded their ability to effectively deliver services, as CHWs performed their duties in addition to income-generating activities.

According to this study, the effectiveness of the Rwanda CHW system was hampered by an irregular system of supervision and trainings. At the individual CHW level, there were varying degrees of capacity noted, and many CHWs did not have an educational background in health prior to delivery of health services. On-the-job trainings were done sporadically, and did not necessarily address specific skills gaps or effectively target the three types of CHWs (ASM, CHSA, *binomes*) at the time of the interviews. The need for standardized, comprehensive training systems was noted across all categories of CHWs, as well as the desire for trainings in self-confidence and communication skills in order to appropriately disseminate information.

In Pakistan, the 100,000 Lady Health Workers, similar to CHWs, each receive 3 months of initial training, and 1 year of supervised fieldwork [19]. Implementing a similar system in Rwanda could address these training concerns. In India, a 12 to 18-month distance-learning course for government and civilian health professionals, the Public Health Resource Network, aims to build health capacity throughout the country. A similar system in Rwanda, that broadly and comprehensively builds capacity for health professionals throughout the country via distance-learning, could be an effective addition to the ongoing capacity-building programmes [20]. A basic preservice training course that provides a minimum set of knowledge and competencies for new recruits would harmonize entry-level CHW knowledge. All CHWs would also benefit from basic and refresher trainings in a wide range of topics, including basic nutrition, feeding practices for infants and young children, management of moderate and severe undernutrition, nutritional assessment, programme management, and monitoring and evaluation.

Additionally, two models that have a professionalized approach for community health workers include the Community Health Agents (CHAs) in Tanzania, and the Health Extension Workers (HEW) in Ethiopia. In Tanzania, the CHAs are formally remunerated, receive 9 months of preservice training, and dually report to health facility staff and village governments [21]. HEWs in Ethiopia receive 1 year of training, are also remunerated by the government, and report to nurses or environmental health professionals at nearby health centres [22]. These formalized CHW systems provide unique models that may have components, such as formalized training and remuneration, worth investigating in Rwanda.

Principles behind Rwanda’s cPBF system, which was only recently implemented at the time of the study, were not well understood by CHWs. The lack of transparency of the process, and confusion around who should receive payments was noted throughout the FGDs. Since this study, additional efforts have been made to strengthen CHW cooperative financial management and understanding of the programme. In 2014, there were 640 CHW cooperatives which primarily delivery health services and generate income through various farm- and food-related activities. Of the net profit collected by these cooperatives, 50% is managed in a fund at the MoH, and is used for CHW trainings and tools, and additional CHW remuneration. To ensure that these cooperatives are managed appropriately, all cooperatives will be required to hire managers by the end of 2015, and a reporting system called the Community Health Workers Financial Tool will be rolled out in 2015 [23]. Further strengthening of the cPBF system, with cooperatives, managers, and advanced tools, will serve to alley some of the anxieties around cPBF support to the CHWs that we identified in this study.

While these improvements have helped to institutionalize cPBF-supported CHW cooperatives, other studies have found higher CHW attrition rates in community financing programmes than in salaried programmes [24, 25]. The long-term sustainability of the Rwandan volunteer programme may prove challenging unless continuous improvements to the cPBF programme are made, and transitioning to an output-based salaried system is being considered with the establishment of well-functioning cPBF at health posts.

Since this study was conducted in 2011, the CHW system has been significantly reorganized and strengthened. In 2012, the MoH and the Ministry of Local Government removed the CHWs in charge of social affairs (CHSA). Each village now has a pair of CHWs (*binomes*) and one ASM who manage maternal and newborn health. In addition to the strengthening of the cPBF system as mentioned above, comprehensive trainings, focusing on capacity building, have also been conducted for all CHWs throughout the country [23].

This study has a number of limitations. Methodologically, quantitative data were not available to complement the qualitative analyses. Additionally, data collection occurred in 2011, before significant changes in the Rwanda CHW system were implemented. Furthermore, while our beneficiary FGDs were composed of females as a result of the larger Government of Rwanda capacity assessment goals, future research investigating both male and female beneficiaries’ perceptions would be a useful addition to the literature. Despite these limitations, we believe that this study provides a unique vantage point into an evolving CHW system, and has the potential to inform future policy decisions as the programme continues to improve.

## Conclusion

While a CHW system can address some of the problems created by a severe human resource shortage, there is a continuous need to harmonize and upgrade the capacity of the CHWs so that they are able to deliver services efficiently and effectively. Both comprehensive and targeted trainings are necessary to fill gaps in knowledge, and must be in conjunction with sufficient follow-up and refresher courses. Targeted trainings based on needs assessments, with proper monitoring, evaluation and supervision, are necessary to maintain the capacities of the CHWs and to scale-up the CHW system. Supervision practices can be maximized, and there is a need to address the issue of institutional incentives that shape the utilization and retention of capacities, which is vital for sustainability. Workloads will continue to be a significant challenge in the underresourced system, and are currently overwhelming to the CHWs. Despite these challenges and clear areas in need of improvement, the CHW system is evolving, and the many CHWs in Rwanda providing vital and necessary services to their communities are remaining motivated by their community recognition and respect.

## Abbreviations

AARR: average annual rate of reduction
ASM: *Animatrice de Santé Maternelle*
CFSVA: Comprehensive Food Security and Vulnerability Analysis and Nutrition Survey
CHAs: community health agents
CHW: community health worker
cPBF: community performance-based financing
FGD: focus group discussion
HEWs: health extension workers
IQR: interquartile
MoH: Ministry of Health
MWH: maternal health worker
CHSA: in charge of social affairs
SD: standard deviation.
